# Supplementary material for: The effect of combining antibiotics on resistance: A systematic review and meta-analysis
Source: eLife. 2024 Dec 20;13:RP93740. doi: 10.7554/eLife.93740 (PMC11661791; doi:10.7554/eLife.93740)
Supplement: Supplementary file 1. — The underlined antibiotics indicate that resistance measurements were made for this antibiotic, reported and extractable from the studies. Justification for resistance outcome extraction is given in Appendix 3—table 1. [file elife-93740-supp1.docx]

**Supplementary file 1.** Overview of the 42 RCTs or quasi-RCTs included in the systematic review and meta-analysis. The underlined antibiotics indicate that resistance measurements were made for this antibiotic, reported and extractable from the studies. Justification for resistance outcome extraction is given in Appendix 3 – table 1.

| Study | Type Of Study | Focused pathogen/ Reason for antibiotic treatment | | Objective(S) | Antibiotics used in Study arms | | Explicit Definition Of Resistance Outcome | Secondary Outcomes extracted |  |
| --- | --- | --- | --- | --- | --- | --- | --- | --- | --- |
|  |  |  |  | | Less antibiotics | More Antibiotics |  |  |  |
| Bender et al. (1979) | RCT | Infection prophylaxis for patients with acute leukemia or malignant lymphomas receiving remission induction chemotherapy | Tolerance, suppression of microbial flora, protection against colonisation and infection | | Gentamicin | Gentamicin, and vancomycin | no | All-cause mortality |  |
| Black et al. (1982) | RCT | enterotoxigenic *Escherichia coli* (ETEC) | Compare two treatments options against ECET. | | Trimethoprim | Trimethoprim, and sulfamethoxazole | no | - |  |
| Chaisson et al. (1997) | RCT | MAC | Safety and activity | | Clarithromycin, and ethambutol | Clarithromycin, ethambutol, and clofazimine | no | All-cause mortality |  |
| Cometta et al. (1994) | RCT | Nosocomial pneumonia, nosocomial sepsis, or severe diffuse peritonitis | Clinical efficacy and tolerance, emergence of resistance and risk of superinfection | | Imipenem | Imipenem, and netilmicin | no | Mortality attributable to infection,  treatment failure, treatment failure due to a change of resistance against the study drugs |  |
| Dawson et al. (2015) | RCT | Mtb | Efficacy, and safety | | Moxifloxacin, pretomanid, and pyrazinamide | Isoniazid, rifampicin, pyrazinamide, and ethambutol | no | Proportion of patients with alterations of the prescribed treatment due to adverse events |  |
| Dekker et al. (1987) | RCT | Prophylaxis for acute nonlymphocytic or lymphocytic leukemia | Efficacy of protecting against infections | | Ciprofloxacin | Trimethoprim, and sulfamethoxazole | no | All-cause mortality, mortality attributable to infection, acquisition of resistance against non-administered antibiotics |  |
| (Dickstein et al., 2020) | RCT | Carbapenem resistant, colistin-susceptible, and gram-negative infections | Development of colistin resistance (secondary outcome of a clinical trial) | | Colistin | Colistin, and meropenem | yes | All-cause mortality, proportion of patients with alterations of the prescribed treatment due to adverse events |  |
| Dubé et al. (1997) | RCT | MAC | Risk of recrudescent MAC bacteraemia, emergence of resistance to clarithromycin. | | Clarithromycin, and clofazimine | Clarithromycin, clofazimine, and ethambutol | no | All-cause mortality, proportion of patients with alterations of the prescribed treatment due to adverse events |  |
| Durante-Mangoni et al. (2013) | RCT | Extensively drug resistant *Acinetobacter baumannii* | Mortality | | Colistin | Colistin, and rifampicin | yes | All-cause mortality, mortality attributable to infection, treatment failure |  |
| Fournier et al. (1999) | RCT | MAC | Efficacy and tolerance. | | Clarithromycin, ethambutol | Clarithromycin, ethambutol, and clofazimine | no | All-cause mortality, proportion of patients with alterations of the prescribed treatment due to adverse events |  |
| Gerecht et al. (1989) | RCT | Cholangitis | Compare a single drug treatment to a two-drug treatment. | | Mezlocillin | Ampicillin, and gentamicin | yes | All-cause mortality, treatment failure as reported in each study, treatment failure due to a change of resistance against the study drugs |  |
| Gibson et al. (1989) | RCT | Febrile neutropenia | Efficacy and side effects | | Ceftazidime | Azlocillin, and amikacin | no | All-cause mortality, mortality attributable to infection, proportion of patients with alterations of the prescribed treatment due to adverse events, acquisition of resistance against non-administered antibiotics, emergence of resistance against non-administered antibiotics |  |
| Haase et al. (1984) | RCT | UTI | Efficacy, tolerance, and safety | | Norfloxacin | Trimethoprim, and sulfamethoxazole | no | Treatment failure, acquisition of resistance against non-administered antibiotics |  |
| Harbarth et al. (2015) | RCT | MRSA | Assess the non-inferiority of a multiple drug treatment in comparison of a single drug treatment. | | Linezolid | Trimethoprim, sulfamethoxazole, and rifampicin | no | All-cause mortality, mortality attributable to infection, treatment failure |  |
| Hodson et al. (1987) | RCT | Cystic fibrosis patients with *P. aeruginosa* | Compare an oral one drug treatment to an intravenous two drug treatment. | | Ciprofloxacin | Azlocillin, and gentamicin | no | - |  |
| Hoepelman et al. (1988) | RCT | Serious bacterial infections | Emergence of resistance of fecal flora | | Ceftriaxone | Cefuroxime, and gentamicin | no | Proportion of patients with alterations of the prescribed treatment due to adverse events |  |
| Hultén et al. (1997) | RCT | *H. pylori* | Antibacterial efficacy, emergence of clarithromycin resistance. | | Clarithromycin | Clarithromycin, and lymecycline | no | - |  |
| Iravani et al. (1981) | RCT | Acute UTI | Efficacy, treatment effects on fecal flora, resistance emergence in the infecting pathogen | | Nalidixic acid | Trimethoprim, and sulfamethoxazole | no | - |  |
| Jacobs et al. (1993) | RCT | Bacterial infections in neutropenic children | Efficacy and safety, tolerance, emergence of resistance and risk of superinfection | | Ceftazidime | Ceftazidime, and tobramycin | yes | All-cause mortality, treatment failure, treatment failure due to a change of resistance against the study drugs |  |
| Jo et al. (2021) | RCT | Determine impact of antibiotics on healthy skin microbiota | Investigate short and long term of the skin microbiome | | Doxcycycline | Trimethoprim, and sulfamethoxazole | no | - |  |
| Macnab et al. (1994) | Quasi-RCT | Mtb | Efficacy, primary drug resistance, bacteriological conversion rates, compliance, and side effects | | Isoniazid, and rifampicin | Isoniazid, rifampicin, and ethambutol | no | Proportion of patients with alterations of the prescribed treatment due to adverse event |  |
| Markowitz et al. (1992) | RCT | *S. aureus* | Efficacy and safety | | Vancomycin | Trimethoprim, and sulfamethoxazole | no | All-cause mortality, treatment failure,  proportion of patients with alterations of the prescribed treatment due to adverse events |  |
| Mavromanolakis et al. (1997) | RCT | Recurrent UTIs | Effect on the aerobic bowel flora, frequency of resistant strains in the fecal flora during and after treatment. | | Norfloxacin, or Nitrofurantoin | Trimethoprim, and sulfamethoxazole | no | - |  |
| May et al. (1997) | RCT | MAC | Clinical and bacteriological efficacy, safety, tolerability | | Clarithromycin, and clofazimine | Clarithromycin, rifabutin, and ethambutol | no | All-cause mortality, treatment failure, proportion of patients with alterations of the prescribed treatment due to adverse events |  |
| McCarty et al. (1988) | RCT | Cystic fibrosis patients with *P. aeruginosa* | Safety, pharmacokinetics of a high-dose singe drug treatment, the effectiveness | | Piperacillin | Piperacillin, and tobramycin | no | All-cause mortality, mortality attributable to infection |  |
| Menon et al. (1986) | RCT | Acute UTI | Efficacy, selection of resistance in Enterobacteriaceae | | Trimethoprim | Trimethoprim, and sulfamethoxazole | no | Acquisition of resistance against non-administered antibiotics |  |
| Miehlke et al. (1998) | RCT | *H. pylori* | Effectiveness, tolerability | | Amoxicillin | Clarithromycin, and metronidazole | no | Proportion of patients with alterations of the prescribed treatment due to adverse events |  |
| Parras et al. (1995) | RCT | MRSA | Efficacy to eradicate, safety | | Mupirocin | Sodium fusidate, trimethoprim, and sulfamethoxazole | no | All-cause mortality, proportion of patients with alterations of the prescribed treatment due to adverse events |  |
| Parry et al. (1977) | Quasi-RCT | *Pulmonary infection* | Effectiveness, treatment failure, treatment success, frequency of ticarcillin resistant organisms, influence of resistance on disease development | | Ticarcillin | Ticarcillin, and gentamicin | no | - |  |
| Parry et al. (2007) | RCT | Multidrug resistant typhoid fever | Efficacy | | Ofloxacin, or Azithromycin | Ofloxacin, and Azithromycin | no | Treatment failure |  |
| Paul et al. (2015) | RCT | MRSA | Test whether a two-drug treatment is non-inferior to a two-drug treatment. | | Vancomycin | Trimethoprim, and sulfamethoxazole | yes | All-cause mortality, treatment failure, acquisition of resistance against non-administered antibiotics, emergence of resistance against non-administered antibiotics | |
| Pogue et al. (2021) | RCT | Gram negative resistant bloodstream infections or pneumonia | Assess superiority of a combination of colistin to monotherapy. | | Colistin | Colistin, and meropenem | yes | All-cause mortality, treatment failure |  |
| Pujol et al. (2021) | RCT | MRSA | Assess treatment success. | | Daptomycin | Daptomycin, and fosfomycin | yes | All-cause mortality, treatment failure, proportion of patients with alterations of the prescribed treatment due to adverse events |  |
| Rubinstein et al. (1995) | RCT | Gram-negative hospital acquired infections | Efficacy, safety | | Ceftazidime | Ceftriaxone, and Tobramycin | yes | All-cause mortality, mortality attributable to infection, treatment failure, proportion of patients with alterations of the prescribed treatment due to adverse events |  |
| Schaeffer et al. (1981) | RCT | UTI | Effectiveness, safety, incidence of resistance in faecal and vaginal flora before, and after treatment. | | Cinoxacin | Trimethoprim, and sulfamethoxazole | no | Proportion of patients with alterations of the prescribed treatment due to adverse events |  |
| Schaeffer and Sisney (1985) | RCT | UTI | Effectiveness, safety, incidence of resistance in fecal and vaginal flora before, and after treatment. | | Norfloxacin | Trimethoprim, and sulfamethoxazole | no | - |  |
| Smith et al. (1999) | RCT | P. aeruginosa | Efficacy | | Azlocillin | Azlocillin, and tobramycin | no | Acquisition of resistance against non-administered antibiotics, emergence of resistance against non-administered antibiotics |  |
| Stack et al. (1998) | RCT | *H. pylori* | Efficacy, safety | | Clarithromycin | Clarithromycin, and metronidazole, or amoxycillin | no | - |  |
| Walsh et al. (1993) | RCT | MRSA | Efficacy of eradication, emergence of resistance, safety | | Novobiocin, and rifampicin | Rifampicin, trimethoprim, and sulfamethoxazole | yes | Treatment failure, acquisition of resistance against non-administered antibiotics, emergence of resistance against non-administered antibiotics |  |
| Winston et al. (1986) | RCT | Prophylaxis for hematological malignancy patients | Efficacy and safety | | Norfloxacin | Vancomycin, and polymyxin | no | Mortality attributable to infection |  |
| Winston et al. (1990) | RCT | Prophylaxis for hematological malignancy patients | Efficacy and safety | | Ofloxacin | Vancomycin, and polymyxin | no | - |  |
| Wurzer et al. (1997) | RCT | *H. pylori* | Effectiveness, emergence of resistance. | | Clarithromycin | Clarithromycin, and amoxycillin | no | - |  |

**References**

Bender, J.F., Schimpff, S.C., Young, V.M., Fortner, C.L., Brouillet, M.D., Love, L.J., and Wiernik, P.H. (1979). Role of vancomycin as a component of oral nonabsorbable antibiotics for microbial suppression in leukemic patients. Antimicrobial agents and chemotherapy *15*, 455‐460.

Black, R.E., Levine, M.M., Clements, M.L., Cisneros, L., and Daya, V. (1982). Treatment of experimentally induced enterotoxigenic Escherichia coli diarrhea with trimethoprim, trimethoprim-sulfamethoxazole, or placebo. Reviews of infectious diseases *4*, 540‐545.

Chaisson, R.E., Keiser, P., Pierce, M., Fessel, W.J., Ruskin, J., Lahart, C., Benson, C.A., Meek, K., Siepman, N., and Craft, J.C. (1997). Clarithromycin and ethambutol with or without clofazimine for the treatment of bacteremic Mycobacterium avium complex disease in patients with HIV infection. AIDS (london, england) *11*, 311‐317.

Cometta, A., Baumgartner, J.D., Lew, D., Zimmerli, W., Pittet, D., Chopart, P., Schaad, U., Herter, C., Eggimann, P., and Huber, O. (1994). Prospective randomized comparison of imipenem monotherapy with imipenem plus netilmicin for treatment of severe infections in nonneutropenic patients. Antimicrobial agents and chemotherapy *38*, 1309‐1313.

Dawson, R., Diacon, A.H., Everitt, D., van Niekerk, C., Donald, P.R., Burger, D.A., Schall, R., Spigelman, M., Conradie, A., Eisenach, K., and et al. (2015). Efficiency and safety of the combination of moxifloxacin, pretomanid (PA-824), and pyrazinamide during the first 8 weeks of antituberculosis treatment: a phase 2b, open-label, partly randomised trial in patients with drug-susceptible or drug-resistant pulmonary tuberculosis. Lancet (london, england) *385*, 1738‐1747. 10.1016/s0140-6736(14)62002-x.

Dekker, A.W., Rozenberg-Arska, M., and Verhoef, J. (1987). Infection prophylaxis in acute leukemia: a comparison of ciprofloxacin with trimethoprim-sulfamethoxazole and colistin. Annals of internal medicine *106*, 7‐11.

Dickstein, Y., Lellouche, J., Schwartz, D., Nutman, A., Rakovitsky, N., Dishon Benattar, Y., Altunin, S., Bernardo, M., Iossa, D., Durante-Mangoni, E., et al. (2020). Colistin Resistance Development Following Colistin-Meropenem Combination Therapy vs. Colistin Monotherapy in Patients with Infections Caused by Carbapenem-Resistant Organisms. Clinical infectious diseases : an official publication of the Infectious Diseases Society of America. 10.1093/cid/ciz1146.

Dubé, M.P., Sattler, F.R., Torriani, F.J., See, D., Havlir, D.V., Kemper, C.A., Dezfuli, M.G., Bozzette, S.A., Bartok, A.E., Leedom, J.M., et al. (1997). A randomized evaluation of ethambutol for prevention of relapse and drug resistance during treatment of Mycobacterium avium complex bacteremia with clarithromycin-based combination therapy. Journal of Infectious Diseases *176*, 1225-1232.

Durante-Mangoni, E., Signoriello, G., Andini, R., Mattei, A., De Cristoforo, M., Murino, P., Bassetti, M., Malacarne, P., Petrosillo, N., Galdieri, N., and et al. (2013). Colistin and rifampicin compared with colistin alone for the treatment of serious infections due to extensively drug-resistant Acinetobacter baumannii: a multicenter, randomized clinical trial. Clinical infectious diseases *57*, 349‐358. 10.1093/cid/cit253.

Fournier, S., Burguière, A.M., Flahault, A., Vincent, V., Treilhou, M.P., and Eliaszewicz, M. (1999). Effect of adding clofazimine to combined clarithromycin-ethambutol therapy for Mycobacterium avium complex septicemia in AIDS patients. European journal of clinical microbiology & infectious diseases *18*, 16‐22.

Gerecht, W.B., Henry, N.K., Hoffman, W.W., Muller, S.M., LaRusso, N.F., Rosenblatt, J.E., and Wilson, W.R. (1989). Prospective randomized comparison of mezlocillin therapy alone with combined ampicillin and gentamicin therapy for patients with cholangitis. Archives of internal medicine *149*, 1279‐1284.

Gibson, J., Date, L., Joshua, D.E., Young, G.A., Wilson, A., Benn, R., Benson, W., Iland, H., Vincent, P.C., and Kronenberg, H. (1989). A randomised trial of empirical antibiotic therapy in febrile neutropenic patients with hematological disorders: ceftazidime versus azlocillin plus amikacin. Australian and new zealand journal of medicine *19*, 417‐425.

Haase, D.A., Harding, G.K., Thomson, M.J., Kennedy, J.K., Urias, B.A., and Ronald, A.R. (1984). Comparative trial of norfloxacin and trimethoprim-sulfamethoxazole in the treatment of women with localized, acute, symptomatic urinary tract infections and antimicrobial effect on periurethral and fecal microflora. Antimicrobial agents and chemotherapy *26*, 481‐484.

Harbarth, S., von Dach, E., Pagani, L., Macedo-Vinas, M., Huttner, B., Olearo, F., Emonet, S., and Uçkay, I. (2015). Randomized non-inferiority trial to compare trimethoprim/sulfamethoxazole plus rifampicin versus linezolid for the treatment of MRSA infection. Journal of antimicrobial chemotherapy *70*, 264‐272. 10.1093/jac/dku352.

Hodson, M.E., Roberts, C.M., Butland, R.J., Smith, M.J., and Batten, J.C. (1987). Oral ciprofloxacin compared with conventional intravenous treatment for Pseudomonas aeruginosa infection in adults with cystic fibrosis. Lancet (london, england) *1*, 235‐237.

Hoepelman, I.M., Rozenberg-Arska, M., and Verhoef, J. (1988). Comparative study of ceftriaxone monotherapy versus a combination regimen of cefuroxime plus gentamicin for treatment of serious bacterial infections: the efficacy, safety and effect on fecal flora. Chemotherapy *34 Suppl 1*, 21‐29. 10.1159/000238643.

Hultén, K., Jaup, B., Stenquist, B., and Engstrand, L. (1997). Combination treatment with ranitidine is highly efficient against Helicobacter pylori despite negative impact of macrolide resistance. Helicobacter *2*, 188‐193.

Iravani, A., Richard, G.A., Baer, H., and Fennell, R. (1981). Comparative efficacy and safety of nalidixic acid versus trimethoprim/sulfamethoxazole in treatment of acute urinary tract infections in college-age women. Antimicrobial agents and chemotherapy *19*, 598‐604.

Jacobs, R.F., Vats, T.S., Pappa, K.A., Chaudhary, S., Kletzel, M., and Becton, D.L. (1993). Ceftazidime versus ceftazidime plus tobramycin in febrile neutropenic children. Infection *21*, 223‐228.

Jo, J.H., Harkins, C.P., Schwardt, N.H., Portillo, J.A., Zimmerman, M.D., Carter, C.L., Hossen, M.A., Peer, C.J., Polley, E.C., Dartois, V., et al. (2021). Alterations of human skin microbiome and expansion of antimicrobial resistance after systemic antibiotics. Sci Transl Med *13*, eabd8077. 10.1126/scitranslmed.abd8077.

Macnab, M.F., Bohmer, P.D., and Seager, J.R. (1994). Evaluation of the 3-drug combination, Rifater, versus 4-drug therapy in the ambulatory treatment of tuberculosis in Cape Town. South African medical journal *84*, 325‐328.

Markowitz, N., Quinn, E.L., and Saravolatz, L.D. (1992). Trimethoprim-sulfamethoxazole compared with vancomycin for the treatment of Staphylococcus aureus infection. Annals of internal medicine *117*, 390‐398.

Mavromanolakis, E., Maraki, S., Samonis, G., Tselentis, Y., and Cranidis, A. (1997). Effect of norfloxacin, trimethoprim-sulfamethoxazole and nitrofurantoin on fecal flora of women with recurrent urinary tract infections. Journal of chemotherapy (florence, italy) *9*, 203‐207. 10.1179/joc.1997.9.3.203.

May, T., Brel, F., Beuscart, C., Vincent, V., Perronne, C., Doco-Lecompte, T., Saint-Marc, T., Dautzenberg, B., and Grosset, J. (1997). Comparison of combination therapy regimens for treatment of human immunodeficiency virus-infected patients with disseminated bacteremia due to Mycobacterium avium. ANRS Trial 033 Curavium Group. Agence Nationale de Recherche sur le Sida. Clinical infectious diseases *25*, 621‐629.

McCarty, J.M., Tilden, S.J., Black, P., Craft, J.C., Blumer, J., Waring, W., and Halsey, N.A. (1988). Comparison of piperacillin alone versus piperacillin plus tobramycin for treatment of respiratory infections in children with cystic fibrosis. Pediatric pulmonology *4*, 201‐204.

Menon, R., Roberts, F.E., Barr, K.W., Howard, H., Lord, V.L., Hegarty, M.A., Luxton, D.E., and Lacey, R.W. (1986). Comparison of a slow-release trimethoprim with co-trimoxazole: efficacy and selection of resistance in the Enterobacteriaceae. Journal of antimicrobial chemotherapy *18*, 415‐420.

Miehlke, S., Meining, A., Lehn, N., Höchter, W., Weingart, J., Simon, T., Krämer, W., Klann, H., Bolle, K., Sommer, A., and et al. (1998). Comparison of omeprazole, metronidazole and clarithromycin with omeprazole/amoxicillin dual-therapy for the cure of Helicobacter pylori infection. Digestion *59*, 646‐650. 10.1159/000007569.

Parras, F., Guerrero, M.C., Bouza, E., Blázquez, M.J., Moreno, S., Menarguez, M.C., and Cercenado, E. (1995). Comparative study of mupirocin and oral co-trimoxazole plus topical fusidic acid in eradication of nasal carriage of methicillin-resistant Staphylococcus aureus. Antimicrobial agents and chemotherapy *39*, 175‐179.

Parry, C.M., Ho, V.A., Phuong le, T., Bay, P.V., Lanh, M.N., Tung le, T., Tham, N.T., Wain, J., Hien, T.T., and Farrar, J.J. (2007). Randomized controlled comparison of ofloxacin, azithromycin, and an ofloxacin-azithromycin combination for treatment of multidrug-resistant and nalidixic acid-resistant typhoid fever. Antimicrobial agents and chemotherapy *51*, 819‐825. 10.1128/aac.00447-06.

Parry, M.F., Neu, H.C., Merlino, M., Gaerlan, P.F., Ores, C.N., and Denning, C.R. (1977). Treatment of pulmonary infections in patients with cystic fibrosis: a comparative study of ticarcillin and gentamicin. J Pediatr *90*, 144-148.

Paul, M., Bishara, J., Yahav, D., Goldberg, E., Neuberger, A., Ghanem-Zoubi, N., Dickstein, Y., Nseir, W., Dan, M., and Leibovici, L. (2015). Trimethoprim-sulfamethoxazole versus vancomycin for severe infections caused by meticillin resistant Staphylococcus aureus: randomised controlled trial. BMJ (clinical research ed.) *350*, h2219. 10.1136/bmj.h2219.

Pogue, J.M., Rybak, M.J., Stamper, K., Marchaim, D., Thamlikitkul, V., Carmeli, Y., Chiu, C., Daikos, G.L., Dhar, S., Mangoni, E.D., et al. (2021). Emergence of Colistin Resistance in the OVERCOME Trial: Impact of Combination Therapy with Meropenem. Open Forum Infectious Diseases *8*, S418-S419. 10.1093/ofid/ofab466.828.

Pujol, M., Miró, J.M., Shaw, E., Aguado, J.M., San-Juan, R., Puig-Asensio, M., Pigrau, C., Calbo, E., Montejo, M., Rodriguez-Álvarez, R., et al. (2021). Daptomycin Plus Fosfomycin Versus Daptomycin Alone for Methicillin-resistant Staphylococcus aureus Bacteremia and Endocarditis: A Randomized Clinical Trial. Clin Infect Dis *72*, 1517-1525. 10.1093/cid/ciaa1081.

Rubinstein, E., Lode, H., Grassi, C., Castelo, A., Ward, K., Alanko, K., Hauck, A., Herden, A., Hoffman, P., Inthun, A., et al. (1995). Ceftazidime monotherapy vs. Ceftriaxone/tobramycin for serious hospital- acquired gram-negative infections. Clinical Infectious Diseases *20*, 1217-1228.

Schaeffer, A.J., Flynn, S., and Jones, J. (1981). Comparison of cinoxacin and trimethoprim-sulfamethoxazole in the treatment of urinary tract infections. Journal of urology *125*, 825‐827.

Schaeffer, A.J., and Sisney, G.A. (1985). Efficacy of norfloxacin in urinary tract infection biological effects on vaginal and fecal flora. Journal of urology *133*, 628‐630.

Smith, A.L., Doershuk, C., Goldmann, D., Gore, E., Hilman, B., Marks, M., Moss, R., Ramsey, B., Redding, G., Rubio, T., et al. (1999). Comparison of a β-lactam alone versus β-lactam and an aminoglycoside for pulmonary exacerbation in cystic fibrosis. Journal of Pediatrics *134*, 413-421.

Stack, W.A., Knifton, A., Thirlwell, D., Cockayne, A., Jenkins, D., Hawkey, C.J., and Atherton, J.C. (1998). Safety and efficacy of rabeprazole in combination with four antibiotic regimens for the eradication of Helicobacter pylori in patients with chronic gastritis with or without peptic ulceration. American journal of gastroenterology *93*, 1909‐1913. 10.1111/j.1572-0241.1998.00582.x.

Walsh, T.J., Standiford, H.C., Reboli, A.C., John, J.F., Mulligan, M.E., Ribner, B.S., Montgomerie, J.Z., Goetz, M.B., Mayhall, C.G., and Rimland, D. (1993). Randomized double-blinded trial of rifampin with either novobiocin or trimethoprim-sulfamethoxazole against methicillin-resistant Staphylococcus aureus colonization: prevention of antimicrobial resistance and effect of host factors on outcome. Antimicrobial agents and chemotherapy *37*, 1334‐1342.

Winston, D.J., Ho, W.G., Bruckner, D.A., Gale, R.P., and Champlin, R.E. (1990). Ofloxacin versus vancomycin/polymyxin for prevention of infections in granulocytopenic patients. American journal of medicine *88*, 36‐42.

Winston, D.J., Ho, W.G., Nakao, S.L., Gale, R.P., and Champlin, R.E. (1986). Norfloxacin versus vancomycin/polymyxin for prevention of infections in granulocytopenic patients. Am J Med *80*, 884-890. 10.1016/0002-9343(86)90633-9.

Wurzer, H., Rodrigo, L., Stamler, D., Archambault, A., Rokkas, T., Skandalis, N., Fedorak, R., Bazzoli, F., Hentschel, E., Mora, P., and et al. (1997). Short-course therapy with amoxycillin-clarithromycin triple therapy for 10 days (ACT-10) eradicates Helicobacter pylori and heals duodenal ulcer. ACT-10 Study Group. Alimentary pharmacology & therapeutics *11*, 943‐952.
